# Four New Genes of Cyanobacterium *Synechococcus elongatus* PCC 7942 Are Responsible for Sensitivity to 2-Nonanone

**DOI:** 10.3390/microorganisms8081234

**Published:** 2020-08-13

**Authors:** Olga A. Koksharova, Alexandra A. Popova, Vladimir A. Plyuta, Inessa A. Khmel

**Affiliations:** 1 Belozersky Institute of Physico-Chemical Biology, Lomonosov Moscow State University, Leninskie Gory, 1-40, 119992 Moscow, Russia; 2 Institute of Molecular Genetics of National Research Center “Kurchatov Institute”, Kurchatov Square, 2, 123182 Moscow, Russia; alexandra.a.popova@gmail.com (A.A.P.); plyutaba@gmail.com (V.A.P.); khmel@img.ras.ru (I.A.K.); 3 Winogradsky Institute of Microbiology, The Federal Research Centre “Fundamentals of Biotechnology” of the Russian Academy of Sciences, Prospekt 60 let Oktyabrya, 7/2, 117312 Moscow, Russia

**Keywords:** microorganisms, genetic control, 2-nonanone, VOCs, ABC-transporter, cell membranes, resistant mutants, transposon mutagenesis, DNA restriction-modification, ketone toxicity

## Abstract

Microbial volatile organic compounds (VOCs) are cell metabolites that affect many physiological functions of prokaryotic and eukaryotic organisms. Earlier we have demonstrated the inhibitory effects of soil bacteria volatiles, including ketones, on cyanobacteria. Cyanobacteria are very sensitive to ketone action. To investigate the possible molecular mechanisms of the ketone 2-nonanone influence on cyanobacterium *Synechococcus elongatus* PCC 7942, we applied a genetic approach. After Tn5-692 transposon mutagenesis, several 2-nonanone resistant mutants have been selected. Four different mutant strains were used for identification of the impaired genes (*Synpcc7942_1362, Synpcc7942_0351*, *Synpcc7942_0732, Synpcc7942_0726*) that encode correspondingly: 1) a murein-peptide ligase Mpl that is involved in the biogenesis of cyanobacteria cell wall; 2) a putative ABC transport system substrate-binding proteins MlaD, which participates in ABC transport system that maintains lipid asymmetry in the gram-negative outer membrane by aberrantly localized phospholipids transport from outer to inner membranes of bacterial cells; 3) a conserved hypothetical protein that is encoding by gene belonging to phage gene cluster in *Synechococcus elongatus* PCC 7942 genome; 4) a protein containing the VRR-NUC (virus-type replication-repair nuclease) domain present in restriction-modification enzymes involved in replication and DNA repair. The obtained results demonstrated that 2-nonanone may have different targets in *Synechococcus elongatus* PCC 7942 cells. Among them are proteins involved in the biogenesis and functioning of the cyanobacteria cell wall (*Synpcc7942_1362, Synpcc7942_0351, Synpcc7942_0732*) and protein participating in stress response at DNA restriction-modification level (*Synpcc7942_0726)*. This paper is the first report about the genes that encode protein products, which can be affected by 2-nonanone.

## 1. Introduction

Volatile organic compounds (VOCs) are diverse organic molecules (alcohols, ketones, aldehydes, acids, terpenoids, sulfur-containing compounds, etc.) that are producing by bacteria, fungi, plants, and animals. VOCs play an important role in communication between organisms in natural communities and are considering as “infochemicals” in ecological microbiology by now [1,2,3,4,5,6,7,8,9]. The microbial volatile database “mVOC” is available [2]. The volatiles have a wide range of effects: antimicrobial action, stimulation or suppression of plant growth, animal (nematode and insects) attraction or deterrence [4,5,6,7]. VOCs may participate in the regulation of bacteria cellular processes, in the communication of bacteria and their interaction with higher organisms [3,4,6,8]. Rhizosphere bacteria of *Pseudomonas* and *Serratia* genera are known to produce volatiles providing antagonistic activity of these strains against a number of prokaryotic (heterotrophic bacteria, cyanobacteria) and eukaryotic organisms (fungi, nematodes, *Drosophila*) [5,6,7]. In our previous study, it was found that the ketones 2-nonanone (14.4 ± 5.0%) and 2-undecanone (12.0 ± 3.6%) were in bacterial VOCs mixture [7]. Microbial volatiles ketones demonstrate different types of biological activity [3]. The detailed molecular mechanisms of the effects of ketones on bacteria, fungi, algae, and plants are not known. Due to their high lipophilicity, ketones can easily penetrate membranes of bacteria cells and these organic molecules may modify the membrane chemical structure through nucleophilic addition between carbonyl groups and acetyl amine groups [9]. The permeability for lipophilic molecules like ketones depends on their acyl chain length. So, the biological effects of ketones may depend on the ketone molecule size. Ketones can form Schiff bases with protein amino groups and therefore lead to disruption of normal protein activity [10,11]. It was demonstrated that ketones bind with protein (bovine serum albumin) [12]. Moreover, 2-nonanone and 2-undecanone demonstrated an inhibitory action on protein refolding in bacteria [13], which can suppress the expression of genes involved in Quorum Sensing regulation in bacterial cells [14] and negatively affected biofilm formation by *Agrobacterium tumefaciens* [15].

Strong inhibitory effect of 2-nonanone, 2-heptanone, and 2-undecanone was shown in different taxonomic groups of organisms, including cyanobacteria (*Synechococcus elongatus* PCC 7942, *Anabaena* sp. PCC 7120, *Nostoc* PCC 6310, *Nostoc* sp. PCC 9305), *Drosophila melanogaster* and nematodes [7]. In our experiments [16], the effects of two ketones (2-nonanone and 2-undecanone) on the cyanobacterial photosynthetic apparatus were very similar causing significant disturbances in the photosynthetic apparatus of cyanobacterium *Synechococcus elongatus* PCC 7942 by inhibition of electron transport through photosystem II. Ketones can participate in antagonistic interactions in the environment raising the question about the cellular targets, on which ketones act and about the mechanisms of their action. One of the possible experimental approaches to find an answer to this question is a genetic approach. Ketone-resistant mutants could be useful tools for gene identification and detection of ketone targets in cells.

The main goal of this study was to obtain 2-nonanone resistant mutants of the cyanobacterium *Synechococcus elongatus* PCC 7942 and to identify the corresponding mutated genes. In the present study, 2-nonanone resistant mutants have been obtained by transposon mutagenesis. Transposon insertions were localized in four different genes of *Synechococcus elongatus* PCC 7942.

## 2. Materials and Methods

### 2.1. Bacterial Strains, Plasmids, and Growth Conditions

Bacterial strains and plasmids used in the work are listed in Table 1.

The wild type of *Synechococcus elongatus* PCC 7942 (herein referred to as *Synechococcus*) (originated from the Moscow State University laboratory collection) and its derivative mutants were grown on solid (1.2% Bacto-agar) BG11_N_ medium that contains sodium nitrate or in liquid BG11_N_ medium [17] at 25 °C and an illumination of 50 µmol photons m^−2^ s^−1^. For growing of mutants, antibiotics were added into growth medium at the following concentrations: streptomycin (Sm; 2 μg/mL), spectinomycin (Sp; 10 μg/mL), erythromycin (Em; 10 μg/mL), kanamycin (Km; 50 μg/mL).

*Escherichia coli* strains were cultivated in the liquid Luria-Bertani (LB) medium or on Petri dishes with agarized LB medium (1.5% agar) [18] at a temperature of 37 °C. Antibiotics were added at the following concentrations: streptomycin (Sm; 25 μg/mL), spectinomycin (Sp; 50 μg/mL), erythromycin (Em; 50 μg/mL), kanamycin (Km; 100 μg/mL), ampicillin (Ap; 50 μg/mL).

### 2.2. Transposon Mutagenesis of Synechococcus

Tn5-692 transposon (in plasmid pRL692; GenBank accession no. AF424805) was efficiently used earlier for mutagenesis of *Synechococcus* [19]. Transposon mutagenesis was carried out as we described in [19,22] with some modifications. The transfer of the Tn5-692 transposon into the chromosome of *Synechococcus* was performed by triparental mating of cyanobacterium as a recipient with *E. coli* strains HB101, which harbor the Tn5-692 transposon on the plasmid pRL692 and conjugal plasmid pRL443 used for the mobilization of the plasmid pRL692 (Table 1). *E. coli* strains were grown in 3 mL LB with the appropriate antibiotic(s) and incubated at 37 °C overnight. Cells of *E. coli* were diluted 1:20 and were grown for 1.5–2 h at 37 °C. Then *E. coli* cells were harvested from 1 mL of each bacterial culture by centrifugation and resuspended in 1 mL fresh LB. This step was repeated twice to wash the cells. After the third centrifugation, the cells were resuspended in 100 µL BG11 _N_ medium. These cells were mixed with 100 µL of concentrated 50-fold in BG11 _N_ *Synechococcus* cells that were grown on a rotary shaker for 5 days before mating till OD_λ = 700 nm_ 0.6–0.7. The conjugation mixture was incubated for 1 h in low light (15 µmol photons m^−2^ s^−1^) at 25 °C. Then the cells were spread on sterile nitrocellulose filters (Millipore Corporation, Billerica, MA, USA) laid on BG11 _N_ + 5% (vol/vol) LB agar plates (mating plates). The mating plates were incubated without antibiotic selection for 24 h in low light at 25 °C, and then the filters were transferred to BG11 _N_ plates for 24 h incubation to give time for cyanobacteria to grow and only afterward the filters were transferred to BG11 _N_ plates with Sp (10 μg/mL) and Em (10 μg/mL). Transconjugant colonies were obtained on nitrocellulose filters after 15–20 days under light incubation. In the result of Tn5-692 mutagenesis, more than 2500 transposon mutants have been obtained on a selective medium containing antibiotics Sp, Em (transposon markers).

### 2.3. Selection of Synechococcus Mutants Resistant to 2-Nonanone Action

Transposon mutants of *Synechococcus* were selected in the presence of high quantity (100 μmol) of 2-nonanone (>99% purity, Sigma-Aldrich Chimie GmbH, Steinheim, Germany). Ketone action was assayed as described in [7]. Colonies of mutants were replicated on the plates with agarized BG11 _N_ medium. Wild type cells were used as control. Small aluminum foil boxes were placed on the surfaces of the agar in the center of the Petri dishes and filled with 100 μmol of 2-nonanone; this quantity of ketone completely inhibits the growth of *Synechococcus* [7]. The plates were sealed with two layers of «Parafilm M» (Pechiney Plastic Packaging Company, Chicago, IL, USA). Colonies of mutants were incubated with 2-nonanone no less than a week. Mutants resistant to 2-nonanone (NR-mutants, “Nonanone Resistant”) were selected for further investigations. After several rounds of verification of their growth ability at ketone presence, eleven 2-nonanone resistant mutants of *Synechococcus* have been selected. Four the most stable mutants–NR401(Tn), NR385(Tn), NR365(Tn), and NR359(Tn)–have been chosen for further investigations.

### 2.4. Identification of Transposon Insertion Sites

Genomic DNA was isolated from the wild type and the 2-nonanone resistant mutants of *Synechococcus* by using phenol-chloroform extraction as described in [23]. Genomic DNA from the mutants was completely digested during the night by the *SalI* enzyme according to the manufacturer’s protocol (Fermentas, Thermo Fisher Scientific, Vilnius, Lithuania). The recognition site for *SalI* is absent inside of the transposon Tn5-692. DNA fragments were diluted and self-ligated (self-circularized) with T4 DNA ligase (Fermentas, Thermo Fisher Scientific, Vilnius, Lithuania) and used for the transformation of competent *E. coli* XL-Blue cells. Plasmid DNA was isolated from the *E. coli* transformants resistant to Sm (25 μg/mL), Sp (50 μg/mL), Em (50 μg/mL) as these plasmids were supposed to carry the transposon Tn5-692 and flanking fragments of genomic DNA from the transposon insertion regions. For isolation of plasmid DNA, the GeneJET Plasmid Miniprep Kit (Fermentas, Vilnius, Lithuania) was applied. Isolated plasmids were sequenced with pW-TnR:923 L21 primer (Table 2) that was designed to the end of the transposon Tn5-692.

The sequencing of DNA samples was performed using the ABI PRISM^®^ BigDye™ Terminator v.3.1 reagent kit (Applied Biosystems, ThermoFisher Scientific, Waltham, MA, USA). The reaction products were analyzed on an ABI PRISM 3100-Avant automatic sequencer at the Interinstitutional Center for Collective Use of the “GENOM” IMB RAS (http:www.genome-centre.narod.ru/).

### 2.5. Construction of Knockout-Mutants by Site-Directed Mutagenesis

**Figure 1:**
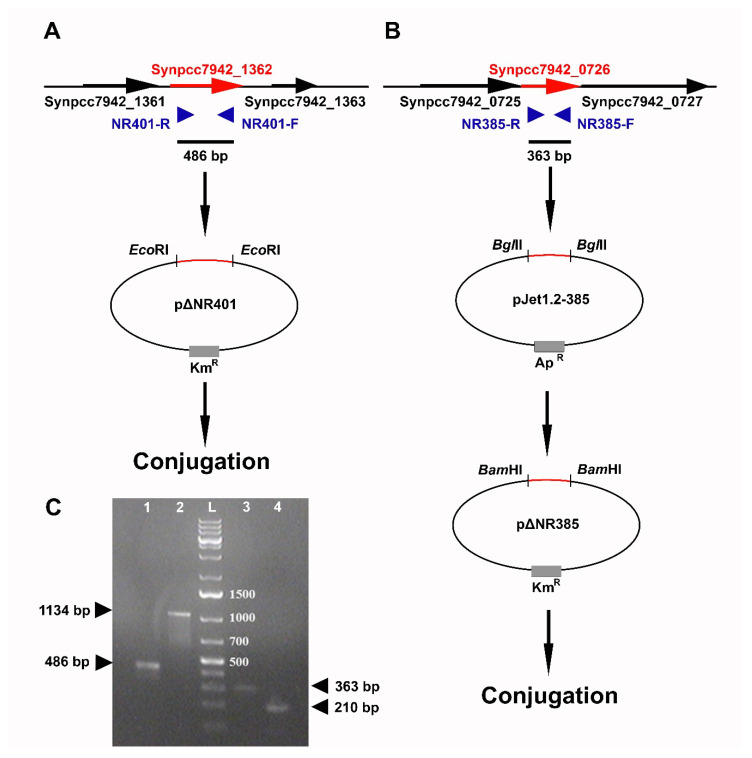
This scheme represents the genetic constructions that have been made by using the deleted copies of the target genes for site-directed mutagenesis of *Synechococcus* wild type cells. (**A**) Construction of a plasmid pΔNR401. Deleted copy of the *Synpcc7942_1362* gene was amplified and cloned into the pRL498 vector in *Eco*RI sites. Blue arrows correspond to primers. (**B**) Construction of a plasmid pΔNR385. Deleted copy of *Synpcc7942_0726* gene was amplified and cloned into pJet1.2/blunt vector. The fragment was digested in *Bgl*II/*Bam*HI sites and cloned into pRL498 for further conjunction. Blue arrows correspond to primers. (**C**) Gel electrophoresis of the PCR products of the target genes. Lanes: **1** *Synpcc7942_1362* (for ΔNR401), **2** *Synpcc7942_0351* (for ΔNR365), **3** *Synpcc7942_0726* (for ΔNR385), and **4** *Synpcc7942_0732* (for ΔNR359), **L**-GeneRuler 1 kb Plus DNA ladder.

Site-directed mutagenesis was performed via homologous recombination of integrative plasmids bearing deleted copies of target genes with chromosomal DNA of the *Synechococcus* wild type. Partial copies of the *Synpcc7942_1362* and *Synpcc7942_0726* genes were amplified from *Synechococcus* DNA (Figure 1) with specific primers (Table 2). PCR was carried out on a Tercik DNA amplifier (DNA Technology, Moscow, Russia) by using DreamTaq PCR Master Mix (Fermentas, EU), under the following conditions: (1) with NR401-F/R primers: 95 °C for 2 min, followed by 35 cycles at 95 °C for 30 s, 66 °C for 40 s, 72 °C for 1 min, and the extension step 72 °C for 10 min; (2) with NR385-F/R primers: 95 °C for 2 min, followed by 35 cycles at 95 °C for 30 s, 66 °C for 40 s, 72 °C for 1 min, and the extension step 72 °C for 10 min. All DNA fragments were detected in the 1.5% agarose gel (VWR Life Science AMRESCO, Solon, OH, USA) stained by Ethidium bromide (5 µg/mL) (VWR Life Science AMRESCO, Solon, OH, USA) and irradiated with UV light with a wavelength of 302 nm (ECX-F15.M, Vilber Lourmat Electronic Ballast transilluminator, Merck KGaA, Darmstadt, Germany) and gel documentation system Gel Imager-2, GI-2, Helicon, Moscow, Russia). DNA fragments were purified using the Wizard SV Gel kit (Promega Corp, Madison, WI, USA) and from reaction PCR mixtures by the PCR Clean-Up System kit (Promega, Madison, WI, USA). GeneRuler 1 kb Plus DNA Ladder 75 to 20,000 bp (ThermoFisher Scientific, Waltham, MA, USA) was used for the sizing of DNA fragments during electrophoresis.

PCR product of the deleted fragment (486 bp) of the *Synpcc7942_136*2 gene was directly cloned in the *EcoRI* site of the pRL498 vector (Figure 1A), producing a plasmid pΔNR401 (Table 1). PCR product of the deleted fragment of the *Synpcc7942_0726* (363 bp), firstly, was subcloned into the *BglII* site of pJet1.2/blunt vector (CloneJet PCR Cloning Kit, Fermentas) and afterward, it was cloned into the *BamHI* site of the pRL498 vector (Figure 1B), producing a plasmid pΔNR385 (Table 1).

Plasmid transformation of the competent cells of *E. coli* XL-Blue, selection, and testing of transformants on the medium with Km (50 μg/mL) were performed using standard techniques [24]. Obtained transformants were used for the further tri-parental mating with helper conjugative plasmid pRL443 for the conjugation transfer of pΔNR401 and pΔNR385 plasmids into *Synechococcus* wild type cells. Kanamycin-resistant transconjugants with plasmids integrated into the chromosome due to homologous recombination were selected on BG11_N_ agar plates supplemented with 100 µg/mL kanamycin, they resulting in ΔNR401 (PCC 7942::pΔNR401) and ΔNR385 (PCC 7942::pΔNR385) mutants, correspondingly. To evaluate the resistance of the obtained insertional mutants ΔNR401 and ΔNR385 to 2-nonanone, cells of these mutants were incubated on plates containing 100 µmol 2-nonanone in the gaseous phase.

The diligent cloning of deleted copy of the gene S*ynpcc7942_0351* in *E. coli* cells was not successful due to unknown reasons. In the case of the very small gene *Synpcc7942_0732*, the inactivation has not been performed due to the lack of the sufficient size of this small fragment DNA for recombination.

### 2.6. Light Microscopy

Morphological differences between the wild type of *Synechococcus* and the mutant cells were analyzed by using Zeiss Axiovert 200 M inverted microscope equipped with Plan-Neofluar 100/1.3 oil immersion objective (Carl Zeiss GmbH, Jena, Germany) and supported by AxioVision SE64 Rel. 4.9.1 Software. Cell sizes were analyzed on the images recorded with a CCD-camera AxioCam MRc.5 (Carl Zeiss). The total number of cells counted was no less than 500 cells per sample.

### 2.7. Data Analysis

Sequencing results were analyzed by using software BioEdit 7.2.5. The search for homologous nucleotide sequences was performed using the BLAST program (Basic Local Alignment Search Tool) in the GenBank database of the US National Center for Biotechnology Information (http://www.ncbi.nlm.nih.gov) and the full genome sequence of *Synechococcus elongatus* PCC 7942 (https://www.ncbi.nlm.nih.gov/nuccore/NC_007604.1). Identification of protein conserved domains was performed by NCBI Conserved Domain Database (CDD). Protein–protein interactions were analyzed by STRING (Protein–Protein Interaction Networks Functional Enrichment Analysis; https://string-db.org/ and by KEGG database (http://www.genome.jp/kegg/).

## 3. Results

### 3.1. Localization of Tn5-692 Transposon Insertions in the Resistant Mutants of Synechococcus

**Figure 2:**
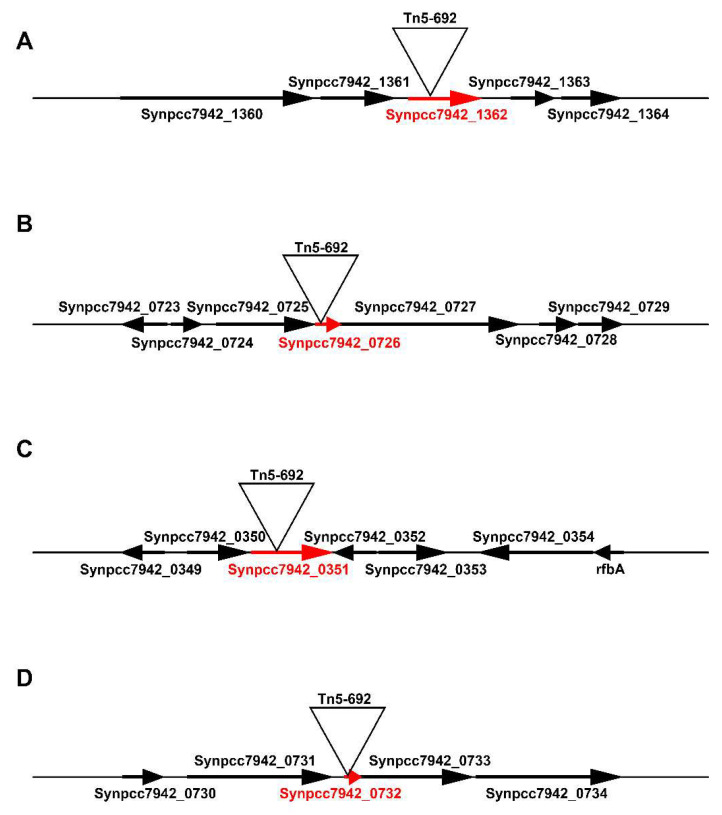
This scheme shows the transposon Tn5-692 localization in genomic regions of *Synechococcus* NR-mutants that were resistant to 2-nonanone. (**A**) Transposon insertion in *Synpcc7942_1362* gene in NR401(Tn) mutant. (**B**) Transposon insertion in *Synpcc7942_0726* gene in NR385(Tn) mutant. (**C**) Transposon insertion in *Synpcc7942_0351* gene in NR365(Tn) mutant. (**D**) Transposon insertion in *Synpcc7942_0732* gene in ΔNR359(Tn) mutant.

Tn5-692 transposon insertions were localized in four different genes (*Synpcc7942_1362*, *Synpcc7942_0726*, *Synpcc7942_0351*, *Synpcc7942_0732*) of the four corresponding mutants (NR401(Tn), NR385(Tn), NR365(Tn), and NR359(Tn) (Figure 2, Table 3).

#### 3.1.1. The *Synpcc7942_1362* Gene Was Inactivated by Tn5-692 in the Mutant NR401(Tn)

**Figure 3:**
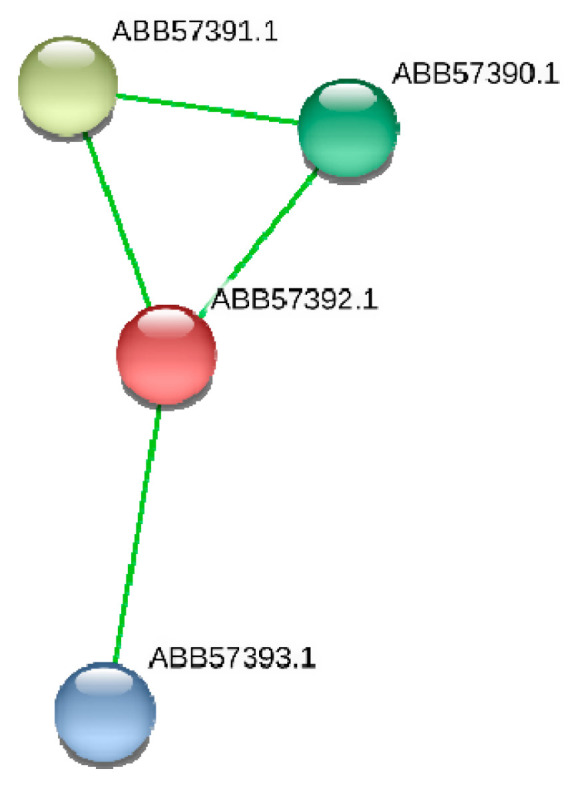
Network of ABB57392.1 protein (*Synpcc7942_1362*) and its protein partners according to STRING (https://string-db.org). In this figure: (**1**) protein ABB57391.1 (*Synpcc7942_1361*) is a conserved hypothetical protein that contains a signal peptide and 8 pentapeptide repeats; (**2**) ABB57390.1 (*Synpcc7942_1360*) is cell envelope-related transcriptional attenuator, polyisoprenyl-teichoic acid--peptidoglycan teichoic acid transferase [EC:2.7.8.-]; (**3**) protein ABB57393.1 (*Synpcc7942_1363*) is uncharacterized protein.

This gene encodes a conserved hypothetical protein (ABB57392.1) of *Synechococcus* with in silico predicted function of murein-peptide ligase (Mpl, UDP-N-acetylmuramate: L-alanyl-gamma-D-glutamyl-meso-diaminopimelate ligase), which is involved in the biogenesis of cyanobacteria cell wall (Table 3). According to STRING (https://string-db.org), hypothetical protein ABB57392.1 (*Synpcc7942_1362*) involves in protein-network (Figure 3) with three other proteins encoding by the its neighboring genes (*Synpcc7942_1360, Synpcc7942_1361, Synpcc7942_1363*) in *Synechococcus* genome. Two of them (ABB57390.1 and ABB57391.1) may be involved in cell envelope metabolism. Namely, the protein ABB57391.1 (*Synpcc7942_1361*) is a conserved hypothetical protein that contains a signal peptide and 8 pentapeptide repeats. The repeats were first identified in HglK, which is required for the localization of heterocyst glycolipids. Other protein, ABB57390.1 (*Synpcc7942_1360*), is cell envelope-related transcriptional attenuator, polyisoprenyl-teichoic acid-peptidoglycan teichoic acid transferase [EC:2.7.8.-], which catalyzes the final step in cell wall teichoic acid biosynthesis. This enzyme participates in the transfer of the anionic cell wall polymers (APs) from their lipid-linked precursor to the cell wall peptidoglycan (PG) (https://www.uniprot.org/uniprot/Q02115).

#### 3.1.2. In the Mutant NR365(Tn) the Transposon Was Inserted in the *Synpcc7942_0351* Gene

**Figure 4:**
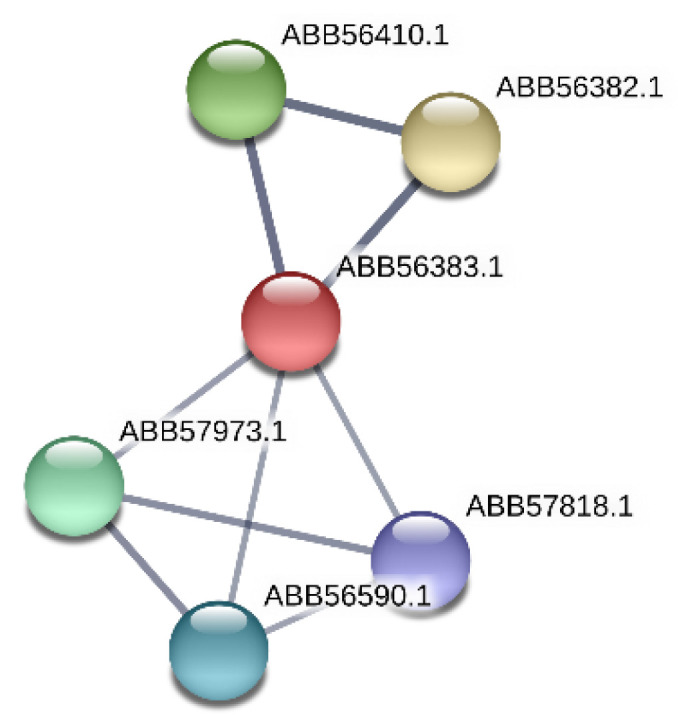
Network of ABB56383.1 protein (*Synpcc7942_0351*) and its protein partners according to STRING (https://string-db.org) is shown. In this figure: (**1**) protein ABB56410.1 (*Synpcc7942_0378*) is MlaE, ABC transporter phospholipid/cholesterol/gamma-HCH transport system permease protein; (**2**) protein ABB56382.1 (*Synpcc7942_0350*) is MlaF, phospholipid/cholesterol/gamma-HCH transport system ATP-binding protein; (**3**) protein ABB57818.1 (*Synpcc7942_1*788) is translocation and assembly module TamB protein; (**4**) protein ABB56590.1 (*Synpcc7942_0558*) is conserved hypothetical protein, chaperone-like protein; (**5**) protein ABB57973.1 (*Synpcc7942_*1943) is cell division protein Ftn2-like.

The product of the *Synpcc7942_0351* gene belongs to the putative ABC transport system substrate-binding proteins MlaD (ABB56383.1), which is similar to ABC transporters providing the resistance to organic solvents (Table 3). It could be also pointed out that protein ABB56383.1 (*Synpcc7942_0351*) forms a network with protein partners (https://string-db.org) (Figure 4). Figure 4 shows such protein network, where two proteins belong to the MlaABCDEF system: the protein ABB56410.1 (*Synpcc7942_0378*) is MlaE, ABC transporter phospholipid/cholesterol/gamma-HCH transport system permease protein and the protein ABB56382.1 (*Synpcc7942_0350*) is MlaF, phospholipid/cholesterol/gamma-HCH transport system ATP-binding protein. Three other proteins are protein ABB57818.1 (*Synpcc7942_1788*), which is translocation and assembly module TamB protein; the protein ABB56590.1 (*Synpcc7942_0558*) is a conserved hypothetical protein, chaperone-like protein and the protein ABB57973.1 (*Synpcc7942_1943*) is cell division protein Ftn2-like, which contains CbpA region. It is known that Curved DNA-binding protein CbpA, contains a DnaJ-like domain, which is characteristic of DnaJ/Hsp40 (heat shock protein 40) proteins. They are highly conserved and play crucial roles in protein translation, folding, unfolding, translocation, and degradation.

#### 3.1.3. In the Mutant NR359(Tn) the Tn5-692 Transposon Was Inserted in the *Synpcc7942_0732* Gene

In the mutant NR359(Tn) the Tn5-692 transposon sequence was flanked by the part of the *Synpcc7942_0732* gene, which is described in the NCBI as encoding a small conserved hypothetical protein (ABB56764.1) of 69 amino acids with an unknown function. The gene belongs to the 23 kb phage gene cluster in *Synechococccus elongatus* PCC 7942 genome (Table 3). In bacterial systems, operon structure often reflects a shared biological function among the protein products of coexpressed genes. Several genes predicted to encode phage proteins, such as the putative phage terminase large subunit (*Synpcc7942_0731*), phage portal protein, lambda (*Synpcc7942_0733*), phage baseplate assembly protein V (*Synpcc7942_0738*), phage P2 protein GPJ (*Synpcc7942_0740*), phage tail protein I (*Synpcc7942_0741*), phage major tail tube protein (*Synpcc7942_0748*), phage tail tape measure protein TP901 (*Synpcc7942_0750*), core region and phage late control D protein GPD (*Synpcc7942_0753*), interspersed by several hypothetical conserved proteins. A lysozyme-encoding gene (*Synpcc7942_0756*) that is present downstream of this region may also be part of this gene cluster.

**Figure 5:**
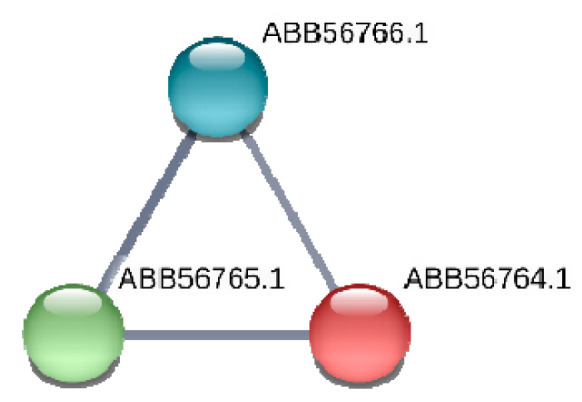
Network of ABB56764.1 protein (*Synpcc7942_0732*) and its protein partners according to STRING (https://string-db.org) is shown. In this figure: protein ABB56765.1 (*Synpcc7942_0733*) is the Phage portal protein, which belongs to the lambda family; protein ABB56766.1 (*Synpcc7942_0734*) is putative DNA primase/helicase.

Moreover, protein ABB56764.1 (*Synpcc7942_0732*) forms network with protein partners (https://string-db.org) (Figure 5). They are encoding by genes that belong to this 23 kb island of phage genes.

#### 3.1.4. The Gene *Synpcc7942_0726* Was Inactivated by Tn5-692 in the Mutant NR385(Tn)

**Figure 6:**
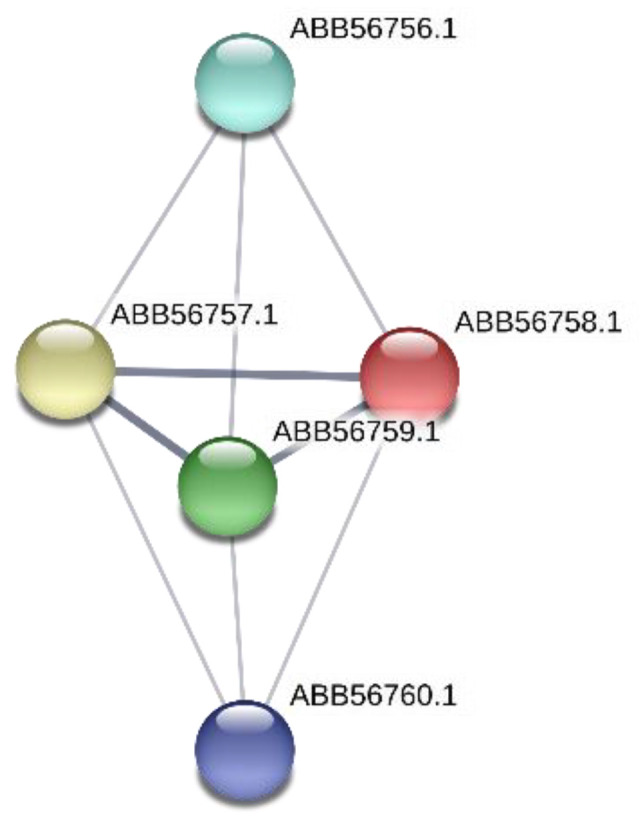
Network of ABB56758.1 protein (*Synpcc7942_0726*) and its protein partners according to STRING (https://string-db.org) is presented. In this figure: the protein ABB56756.1 (*Synpcc7942*_0724) and the protein ABB56760.1 (*Synpcc7942_0728*) are conserved hypothetical proteins; the protein ABB56757.1 (*Synpcc7942_0725*) is DEAD/DEAH box helicase-like enzyme; the protein ABB56759.1 (*Synpcc7942_0727*) is putative DNA primase/helicase.

The gene *Synpcc7942_0726* is predicted to encode a conserved hypothetical protein (ABB56758.1) containing the VRR-NUC (Virus-type Replication-Repair Nuclease) domain. This functional unit is present in the PD-(D/E)XK nuclease superfamily and repair enzymes; these enzymes contain a canonical nuclease catalytic domain typically found in type II restriction endonucleases (Table 3). According to STRING (https://string-db.org), the conserved hypothetical protein ABB56758 participates in protein-network with two other proteins that can be involved in DNA functionality (Figure 6). They are protein ABB56757.1 (*Synpcc7942_0725*) DEAD/DEAH box helicase-like enzyme and protein ABB56759.1 (*Synpcc7942_0727*) that is Mu-like_gpT domain-containing protein. Both protein-partners are encoding by genes that are in the same operon with the gene *Synpcc7942_0726* that was impaired in the mutant NR385(Tn). In this operon (*Synpcc7942_0724-Synpcc7942_0729*) the other genes encode proteins, correspondingly: *Synpcc7942_0724* encodes conserved hypothetical protein; *Synpcc7942_0725* encodes DEAD/DEAH box helicase-like; *Synpcc7942_0727* encodes a conserved hypothetical protein, containing the Zinc-binding domain of primase-helicase domain and topoisomerase-primase (TORPIM) nucleotidyl transferase/hydrolase domain; *Synpcc7942_0728* and *Synpcc7942_0729* are conserved hypothetical proteins.

### 3.2. Directional Inactivation of Synpcc7942_1362 and Synpcc7942_0726 Genes

**Figure 7:**
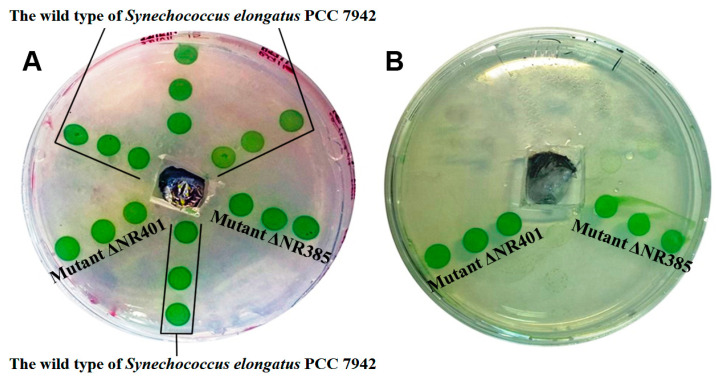
This figure shows the effect of 2-nonanone on the wild type of *Synechococcus* and the insertion of mutants ΔNR401 and ΔNR385. (**A**) Control plate. The growth of the wild type and mutant cells is similar. (**B**) 100 µmol of 2-nononone was added. The growth of the wild type cells is inhibited. The mutants ΔNR401 and ΔNR385 demonstrated growth at the ketone presence.

Mutants ΔNR401 and ΔNR385 with directional inactivation of *Synpcc7942_1362* and *Synpcc7942_0726* genes have been obtained. These mutants demonstrated the same level of resistance to 2-nonanone action (100 µmol) as the corresponding transposon mutants (Figure 7). Moreover, it was found that ΔNR401 and ΔNR385 mutants as well as corresponding transposon mutants were resistant to the 100 µmol of 2-undecanone, revealing cross-resistance of these mutants to this class of VOCs. Mutants ΔNR401 and ΔNR385 demonstrated no difference in growth rate and pigmentation in comparison with the wild type strain.

Thus, the ketone-resistant phenotype due to localization of transposon in the *Synpcc7942_1362* and *Synpcc7942_0726* genes was successfully confirmed by the directed insertional inactivation of these genes.

### 3.3. Morphological Characterization of ΔNR401 and ΔNR385 Mutants

**Figure 8:**
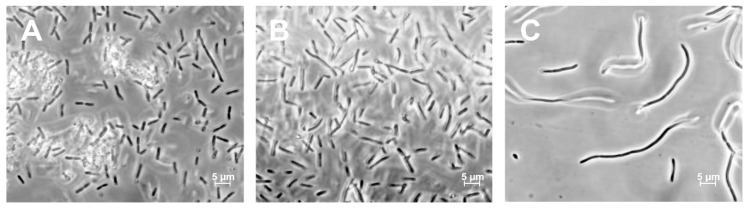
The wild-type and mutant phenotypes of *Synechococcus*. (**A**) Mutant ΔNR385. (**B**) The wild type of *Synechococcus*. (**C**) Mutant ΔNR401.

The average length of cells in the wild type of *Synechococcus* was 3.96 ± 1.62 µm. The mutant ΔNR385 cells showed similar cell sizes (3.95 ± 1.44 µm) (Figure 8A). The significant increase in length was observed in the case of the ΔNR401 mutant cells (Figure 8C). At the same time, there were no significant morphological differences between the mutant ΔNR385 cells (Figure 8A) and the wild type cells (Figure 8B). The mutant ΔNR401 contained three size-types of cells that are visually diverse according to their length with the following sizes–15.7 ± 2.70 µm, 26.93 ± 3.84 µm, and 43.44 ± 2.09 µm (Figure 8C). Thus, microscopic analysis revealed that the ΔNR401 mutant had a greater cell length (up to 4–10 times) in comparison with the wild type of *Synechococcus*.

## 4. Discussion

### 4.1. Three Genes (Synpcc7942_1362, Synpcc7942_0351, Synpcc7942_0732) That Encode Proteins Involved in the Formation, Maintenance, and Functionality of Cyanobacterial Cell Wall

In the DNA of 2-nonanone resistant mutant NR401(Tn) the transposon Tn5-692 was found inserted in the gene *Synpcc7942_1362* encoding conserved hypothetical protein Mpl (murein-peptide ligase, ABB57392.1; https://www.ncbi.nlm.nih.gov/protein/ABB57392.1). By using the site-specific mutagenesis, we have confirmed that disruption of the gene *Synpcc7942_1362* contributes to the resistance of the mutant ΔNR401(Tn) to 2-nonanone.

The *mpl* gene encoding L-alanyl-γ-D-glutamyl-meso-diaminopimelate ligase is well studied in *Escherichia coli*. The product of this gene is involved in the recycling L-alanyl-γ-D-glutamyl-meso-diaminopimelate, which are formed during the degradation of the peptidoglycan layer in bacteria cells during cell division [25]. Compounds formed due to the recycling process constitute 30–60% of the murein layer after the synthesis of a new cell wall in Gram-negative bacteria [26]. Recyclization of murein proceeds in parallel with the functioning of the enzymes MurC, MurD, and MurE, which participate in the biosynthesis of peptidoglycan de novo [27]. When the *mpl* gene was inactivated in the *E. coli* strain, it turned out that the peptidoglycan content in the cells of this mutant did not decrease and that the absence of the product of this gene did not affect cell viability [25]. However, in the absence of murein peptide ligase activity, the content of the peptide triglycan precursor decreases by 50% in the mutant cells, and the products of peptidoglycan degradation presumably accumulate [25]. Therefore, it can be assumed that the presence of these products in the periplasmic space may represent an additional obstacle to the penetration of 2-nonanone molecules into the cytoplasm of cells and thus leads to the resistance of the mutant ΔNR401 cells to the action of this ketone.

It is known that bacterial cell division depends on normal peptidoglycan metabolism and synthesis [26,27,28]. Cell division is mediated by filaments of bacterial tubulin homolog FtsZ and protein FtsA (FtsAZ) that recruit septal peptidoglycan-synthesizing enzymes to the division site [29]. Defects in peptidoglycan synthesis in bacteria lead to elongated cell formation due to a violation of the division process [26]. In our study, we found that the mutant ΔNR401 characterized by long size cells (from15.7 ± 2.70 µm to 43.44 ± 2.09 µm) (Figure 8C) in comparison with the wild type cells (3.96 ± 1.62 µm) of *Synechococcus*. The cell length of the wild type of *Synechococcus* is normally ranged between 2 and 10 µm [30]. That is consistent with the data obtained in this study.

In the mutant NR365(Tn) cells transposon Tn5-692 inactivated a gene *Synpcc7942_0351* that encodes the putative ABC transport system substrate-binding protein (ABB56383.1, https://www.ncbi.nlm.nih.gov/protein/ABB56383.1). This protein has a signal peptide and contains a conserved protein domain family MlaD. MlaD protein belongs to the ABC (ATP-binding cassette) transport system that actively prevents phospholipids accumulation at the cell surface in the absence of extracellular stress [31]. It was proposed that the MlaFEDB complex of ABC transporters is involved in a phospholipid transport pathway that maintains lipid asymmetry in the outer membrane by reverse transport of phospholipids from the outer membrane to the inner membrane in *E. coli* cells [31]. The MlaD protein functions in substrate binding and has a strong affinity for phospholipids and modulates ATP hydrolytic activity of the complex. This substrate-binding protein localizes on the periplasmic face of the inner membrane of *E. coli* cells with an uncleaved signal sequence [31]. Mla mutants of *E. coli* are relatively impermeable to most compounds except SDS-EDTA, and they exhibit no additional defects in lipopolysaccharides or outer membrane protein levels [31]. Phospholipid transport represents an important and so far unsolved problem of Gram-negative bacteria cell envelope biogenesis. New evidence was provided for anterograde phospholipid export by the Mla system [32]. The proposed model indicates that MlaA can both remove phospholipids from the outer membrane external leaflet and deliver them from MlaC into the outer membrane internal leaflet. Phospholipids monitored from the outer membrane external leaflet move in the retrograde direction, while phospholipids transported from the inner membrane move in the anterograde direction [32].

The *Synpcc7942*_*0732* gene was found as a part of the 23 kb phage gene cluster in cyanobacteria chromosome. Cyanobacteria and phages coexist in nature and the gene transfer, loss, and exchange between these organisms are usual events in their co-evolution [33,34,35,36]. It is little known about resistance mechanisms of sea *Synechococcus* strains to phage [36]. We can hypothesize that some of the prophage proteins that are encoding by cyanobacteria genome probably may modify somehow the cell wall of cyanobacteria. In upcoming studies, it will be interesting to analyze the expression of the genes belonging to 23 kb island of phage genes in *Synechococcus* in different physiological conditions, including different stress conditions.

Thus, in three 2-nonanone resistant mutants of cyanobacterium *Synechococcus* transposon Tn5-692 damaged the genes, which encode proteins that could be involved in cell wall biogenesis and functionality. Cell membranes are the first barrier that defenses bacteria cells from different environmental factors and “takes the first blow”. The VOCs are generally lipophilic compounds with high vapor pressure. They freely pass through biological membranes of producers and are released into the atmosphere or in the soil where the producers live. These molecules freely pass also through biological membranes of target organisms and may elicit changes in plasma membrane potential depolarization and activate regulatory proteins [37]. VOCs can interact with and damage lipid membranes [38] and interact with hydrophobic segments in proteins [13,39]. In our study, we found for the first time that ketone 2-nonanone can act on bacterial cells through different targets and by different mechanisms. It can affect peptidoglycan metabolism, ABC transport Mla system activity, and phage-related proteins with unknown functions. Future experiments with the application of transcriptomic and proteomic methods may help to clarify more details of molecular mechanisms of ketone biological action.

### 4.2. The Gene Synpcc7942_0726 Encodes Protein Involved in DNA Metabolism

The gene *Synpcc7942_0726*, inactivated in the mutant NR385(Tn), encodes conserved hypothetical protein ABB56758.1 that possesses the VRR-NUC domain (viral replication and repair (VRR) nuclease (VRR nuc) domain). VRR-Nuc domains were first identified through a combination of sequence homology and secondary structure prediction [40]. It is associated with members of the PD-(D/E)XK nuclease superfamily, which include the type II restriction-modification enzymes [40]. The VRR-NUC domains are common and diverse in the genomes of bacteriophages and prophages and are involved in the replication and recombination of phage chromosomes [41]. Although the functions of the genes encoding the VRR-NUC domains are unknown, most of them are located in operons that include the DNA repair enzymes. Currently, the homologs of the endonuclease FAN1 (KIAA1018) are the only known eukaryotic proteins containing the VRR-NUC domain [42]. The first VRR-NUC domain structures using single-domain proteins derived from three bacteria and bacteriophage have been accomplished [42]. Probably, from these data authors concluded that, at least in bacteria and bacteriophage, VRR-NUC domains resemble Holliday-junction-resolving enzymes both structurally and functionally. The role of these domains in bacteriophage is so far unknown, but by analogy with systems such as λ Rep, T7 endonuclease I, or T4 endonuclease VII it could be suggested that they may be involved in DNA replication, recombination, and packaging [43].

VOCs action on DNA repair [44,45] and gene transcription [46] has been reported for *Escherichia coli*. So, it was shown that *E. coli* strains that lack enzymes in the pathways of DNA repair, DNA metabolic process, and response to stress were highly sensitive to the VOCs of *Muscodor albus* [44]. VOCs permeabilize the bacterial cell membrane and induce disruption of cellular DNA metabolism through DNA damage. Possible mechanisms of VOCs resistance could include maintaining a robust DNA repair pathway, restricting uptake or increasing efflux of VOCs, detoxifying VOCs via modification of its chemical structure, or eliminating affected pathways that trigger cell death [45]. Until now we do not know the exact function of the VRR-NUC domain protein that is encoding by the gene *Synpcc7942_0726* and that was inactivated in the 2-nonanone resistant mutant NR385(Tn) of *Synechococcus*. We hypothesize that mutation in this gene can probably decrease by some unknown mechanism an induction of the RecA-dependent DNA repair and apoptosis induction.

## 5. Conclusions

**Figure 9:**
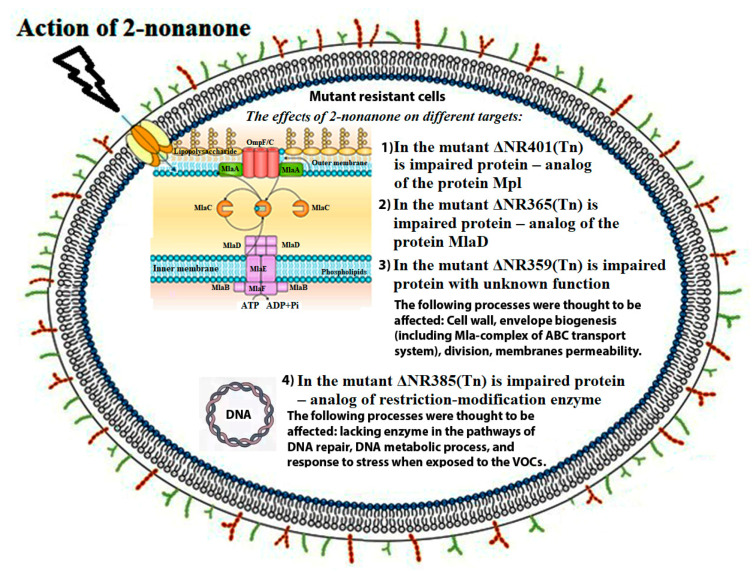
Modes of 2-nonanone action on cyanobacteria cell, (some figure elements are adapted from [47,48]).

In the result of random transposon mutagenesis and a subsequent selection, the four 2-nonanone resistant mutants of *Synechococcus* were chosen for further identification of the impaired genes. Four different genes have been identified. These genes encode proteins with diverse functions and are involved in different cellular processes (Table 3, Figure 9). This observation permits to suggest that the 2-nonanone action on cyanobacteria cells may have several targets and can involve different mechanisms. In our study, we found three genes (*Synpcc7942_1362*, *Synpcc7942_0351*, *Synpcc7942_0732*) that encode proteins probably involved in the formation, maintenance and functionality of cell wall and one gene (*Synpcc7942_0726*) that encodes protein participated in DNA metabolism. Their possible functions and involvement in ketone-resistant mechanisms are discussed above.

This study provides new genetic data that shows the effect of 2-nonanone ketone on multiple targets in cells of the cyanobacterium *Synechococcus*. The biological effects of ketones are pleiotropic, and we are only taking the first steps towards understanding the molecular mechanisms of the complex picture of chemical interactions between organisms using VOCs as communication tools. Information about new genes, mutations in which lead to ketone resistance can also be useful for biotechnological applications in creating bacterial ketone producers. The obtained results allow us to set new tasks for the upcoming research of the molecular mechanisms of action of ketones on bacterial cells.

## Figures and Tables

**Table 1:** Characteristics of bacterial strains and plasmids used in the study.

Strain	Characterization and/or Derivation	Reference or Origin
*Synechococcus elongatus*		
PCC 7942	The wild type	Laboratory collection
NR401(Tn)	Sm ^R^ Sp ^R^ Em ^R^; Tn*5*-692 mutant	This work
NR385(Tn)	Sm ^R^ Sp ^R^Em ^R^; Tn*5*-692 mutant	This work
NR365(Tn)	Sm ^R^ Sp ^R^ Em ^R^; Tn*5*-692 mutant	This work
NR359(Tn)	Sm ^R^ Sp ^R^ Em ^R^; Tn*5*-692 mutant	This work
ΔNR401	Km ^R^; PCC 7942::pΔNR401, transconjugant	This work
ΔNR385	Km ^R^; PCC 7942::pΔNR385, transconjugant	This work
*Escherichia coli*		
XL1-Blue	Host for routine cloning	Collection of the Genetics Department, Moscow State University
HB101 692	HB101 harboring pRL692, Sm ^R^ Sp ^R^ Em ^R^	Laboratory collection
HB101 443	HB101 harboring pRL443, Ap ^R^ Tc ^R^	Laboratory collection
*Plasmids*		
pRL692	Carrying the mobile element Tn5-692, Sm ^R^ Sp ^R^ Em ^R^	[19]
pRL443	Conjugal plasmid, derivative of RP4, Ap ^R^ Tc ^R^; Km ^S^	[20]
pRL498	Km ^R^; plasmid vector for the direct selection	[21]
pJet1.2/blunt	pMB1-ori *bla eco47 IR*::MCS; Ap ^R^	Thermo Fisher Scientific
pJet1.2-385	Ap ^R^; pJet1.2::Δ*Synpcc7942_0726*	This work
pΔNR401	Km ^R^; pRL498::Δ*Synpcc7942_1362*	This work
pΔNR385	Km ^R^; pRL498::Δ*Synpcc7942_0726*	This work

R, resistance; S, sensitivity.

**Table 2:** DNA primers used for the amplification of nucleotide fragments.

Primer Name	Sequence
pW-TnR:946 U21	5′-CTGCTGGCCATTGAGGACACC-3′
pW-TnR:923 L21	5′-CGGGAAACTCCTGAGCCAACT-3′
TN692 R-155	5′-GGCGTTGACATCACTCTG-3′
TN692 F-5485	5′-GTCTAGCTATCGCCATGTAAGC-3′
NR401-F	5′-CCGAATTCGATGCTGTTAGAGG-3′
NR401-R	5′-CCGAATTCGCTTCCAGCTCGAG-3′
NR385-F	5′-CCGAATTCCTCTGGAAGACG-3′
NR385-R	5′-CCGAATTCGCGTCTTGCATC-3′
NR365-F	5′-CCGAATTCGAGAAGGCAGTG-3′
NR365-R	5′-CCGAATTCGAGATCCGTGAC-3′
NR359-F	5′-CCGAATTCGAAGACTTGCAAGC-3′
NR359-R	5′-CCGAATTCGACGGTACTGGATG-3′

NR-primers were used to obtain amplicons that correspond to the deleted copies of the target genes. F—forward primer; R—reverse primer.

**Table 3:** The genes and corresponding proteins, which were impaired in 2-nonanone-resistant mutants of *Synechococcus*.

Mutant	Gene	Protein	Protein Function	Pathway
ΔNR401(Tn)	*Synpcc7942_1362*	conserved hypothetical protein (ABB57392.1)	murein-peptide ligase (Mpl, UDP-N-acetylmuramate: L-alanyl-gamma-D-glutamyl-meso-diaminopimelate ligase)	Biogenesis of cell wall
ΔNR365(Tn)	*Synpcc7942_0351*	putative ABC transport system substrate-binding proteins MlaD (ABB56383.1)	Protein is similar to ABC transporters providing the resistance to organic solvents	Phospholipid transport pathway that maintains lipid asymmetry in the outer membrane
ΔNR359(Tn)	*Synpcc7942_0732*	a small conserved hypothetical protein (ABB56764.1) of 69 amino acids with an unknown function	Gene belonging to phage gene cluster in the *Synechococcus* genome and this protein is involved in interactions with phage proteins	Hypothetically cell membrane modification
ΔNR385(Tn)	*Synpcc7942_0726*	hypothetical protein (ABB56758.1) containing the VRR-NUC domain	Protein contains a Viral replication and repair (VRR) nuclease (VRR nuc) domain. This functional unit is present in the PD-(D/E)XK nuclease superfamily and repair enzymes	DNA metabolism
